# Acknowledgment to Reviewers of *Dermatopathology* in 2021

**DOI:** 10.3390/dermatopathology9010006

**Published:** 2022-01-26

**Authors:** Dermatopathology Editorial Office

**Affiliations:** MDPI AG, St. Alban-Anlage 66, 4052 Basel, Switzerland

Rigorous peer-reviews are the basis of high-quality academic publishing. Due to the great efforts of our reviewers, *Dermatopathology* was able to maintain its standards for the high quality of its published papers. Thanks to the contribution of our reviewers, in 2021, the median time to first decision was 17 days and the median time to publication was 38 days. The editors would like to extend their gratitude and recognition to the following reviewers for their precious time and dedication, regardless of whether the papers they reviewed were finally published:
Arias-Santiago, SalvadorLombardi, TommasoArmesto Alonso, SusanaLosco, LuigiAssaf, ChalidMetze, DieterBahmad, HishamMitteldorf, ChristinaBeltraminelli, HelmutMoertel, ChristopherBentel, Jacqueline M.Moritz, Rose K.C.Bonifaz, AlexandroNambudiri, Vinod E.Bonometti, ArturoNazzaro, GianlucaBroggi, GiuseppeNiebel, DennisBrosens, ErwinNorth, Jeffrey P.Cardoso, José CarlosOsada, Shin-IchiChaber, RadosławPandey, RajeevColmenero, IsabelPaolino, GiovanniColomo, LluísPecorari, GiancarloCota, CarloPicardo, MauroDai, HongyanPuig, LluísEckhart, LeopoldPukhalskaya, TatsianaEisendle, KlausRizzo, AlessandroFeldman, StevenRubin, AdamFernández-Figueras, MaiteRuggiero, AngeloFukasawa, TakemichiSalopek, ThomasGarcía-Pola, María JoséSalvi, MassimoGiovanni, DamianiSantoro, ClaudiaGiubellino, AlessioSavoia, PaolaHay, RoderickShalin, SaraJanik, StefanSlominski, AndrzejKanitakis, JeanSpałek, MateuszKawakami, AkinoriStefanato, CatherineKempf, WernerTaguchi, TakafumiKim, Randie H.Wada, David A.Kiyohara, EijiWassef, MichelLa Placa, MichelangeloWeber, BenediktLateef, ZabeenZavattaro, ElisaLlamas-Velasco, MarZdanowska, NataliaLöffler, Karin U.Zelger, Bernhard

